# Long-term results of Roux-en-Y gastric bypass (RYGB) versus single anastomosis duodeno-ileal bypass (SADI) as revisional procedures after failed sleeve gastrectomy: a systematic literature review and pooled analysis

**DOI:** 10.1007/s00423-024-03557-9

**Published:** 2024-11-23

**Authors:** Theodoros Thomopoulos, Styliani Mantziari, Gaëtan-Romain Joliat

**Affiliations:** 1 Department of Visceral Surgery, Lausanne University Hospital (CHUV), Rue du Bugnon 46, Lausanne, 1011 Switzerland; 2 Faculty of Biology and Medicine, University of Lausanne (UNIL), Lausanne, Switzerland

**Keywords:** Revisional surgery, Sleeve gastrectomy, SADI, Roux-en-Y gastric bypass, Weight loss failure, Functional problems

## Abstract

**Purpose:**

Revisional bariatric surgery (RBS) has flourished during the last decades in many countries, due to suboptimal weight loss or long-term complications of primary bariatric surgery. Restrictive procedures, and in particular sleeve gastrectomy (SG), although widely performed as primary bariatric surgery, seems particularly prone to need surgical revision for the above-mentioned reasons during long-term follow-up. The aim of this systematic review was to compare the long-term (5-year) safety and efficacy between Roux-en-Y gastric bypass (RYGB) and single anastomosis duodeno-ileal bypass (SADI) after ‘failed’ SG.

**Methods:**

A systematic review was performed from 2007 to September 2024. Articles were included if SADI or RYGB were performed as RBS after ‘failed SG’ with follow-up at least 5 years. Pooled analysis was performed to summarize the data.

**Results:**

Among the seven studies eligible and included in this review, the SADI procedure showed comparable results to RYGB in terms of weight loss, nutritional deficiencies, and resolution of comorbidities during the long-term follow-up. However, RYGB proved superior in terms of remission of reflux disease and other functional problems after SG.

**Conclusions:**

The present review found that SADI seems to be a promising and suitable method for suboptimal weight loss after SG, with comparable or even better results to RYGB after 5-year follow-up.

## Introduction

Sleeve gastrectomy (SG) was initially described in 1988 as the restrictive component of the biliopancreatic diversion with a duodenal switch (BPD/DS), which was then performed to add a malabsorptive element to the procedure [1–3]. SG is nowadays the most commonly performed bariatric procedure, due to its relative surgical simplicity and the shorter learning curve compared to Roux-en-Y gastric bypass (RYGB) [4]. SG and RYGB have demonstrated comparable short- and mid-term results in terms of weight loss and improvement of obesity-related comorbidities [5–7]. However, published data have shown that SG may result in insufficient long-term weight loss in 20–50% of patients, especially in the presence of severe obesity (BMI > 50 kg/m^2^) and diabetes mellitus type two (T2DM) [8–10]. Insufficient weight loss represents a frequent indication for revisional bariatric surgery (RBS), aiming to obtain additional weight loss in the long-term. High rates of gastro-esophageal reflux (GERD) have been reported after SG, despite some studies showing initial improvement of reflux related to the weight loss [11, 12]. Indeed, severe GERD after SG represents a frequent indication for RBS, especially in a long-term context [13]. As the number of SG keeps increasing worldwide, bariatric surgeons are increasingly confronted with the necessity of RBS after SG (‘failed’ SG).

There is currently no clear consensus about the ideal RBS procedure for failed SG. Re-sleeve gastrectomy (Re-SG), RYGB, BPD/DS and, since 2007, its omega-loop version called single anastomosis duodeno-ileal bypass (SADI), have been performed in this context. After conversion to RYGB, particularly indicated in patients with SG-related GERD [14], up to 25% of patients still do not manage to obtain optimal weight loss [15, 16]. Another proposed surgical option following a prior unsuccessful SG, is the Re-SG especially in cases where dilatation of the original SG is documented [17], but seems to be inferior in terms of mid- and long-term weight loss outcomes and comorbidity resolution compared to the revisional RYGB [18].

Since SG was originally conceived as the first stage of a planned BPD/DS, the latter remains a valid option not only as a RBS [16, 19] but also as a planned staged two-step procedure with very successful weight loss outcomes [20]. Nevertheless, it needs to be kept in mind that BPD/DS remains a complex procedure, rarely performed nowadays, thus having a longer learning curve and potentially significant complication rates [21]. On the other hand, SADI or SIPS (stomach intestinal pylorus sparing surgery), which is a SADI’s variant, have been rapidly popularized around the world, as they have similar weight loss results to those obtained after the BPD/DS but with less nutritional deficiencies and complications [22]. Although these procedures appear very promising, large series and long-term data to support their efficacy and risk profile are lacking in the literature [23]. In particular, no conclusive evidence exists in the literature as to the efficiency and potential side effects of SADI in comparison to the current gold standard, RYGB.

Lee et al. [24] previously performed a systematic review and meta-analysis, comparing malabsorptive procedures (SADI/BPD-DS group) with the standard RYGB group after failed SG, showing similar results in weight loss and improvement of comorbidities. Moreover, Chierici et al. [25] recently published a systematic review, showing that SADI and one-anastomosis gastric bypass are more effective procedures compared to RYGB/BPD-DS after not only failed SG, but also after other primary restrictive surgeries such as laparoscopic adjustable gastric band and vertical banded gastroplasty. However, these two meta-analyses present some limitations, such as the small sample size (< 20 patients) in most of the included studies, and more importantly, their focus on short/mid-term results (12 to 36 months).

Therefore, the aim of the current systematic review was to focus exclusively on the long-term efficiency of RYGB versus SADI after ‘failed’ SG, with a follow-up of at least 5 years.

## Materials and methods

A comprehensive literature review was conducted on PubMed/MEDLINE, EMBASE, Web of Science, and the Cochrane Central Register of Controlled Trials from 2007 (first description of SADI) until September 2024. Only English-language articles were considered. The search algorithm included free text such as: ‘failed sleeve gastrectomy’, ‘revisional surgery’, ‘treatment failure’, ‘weight recidivism’, ‘Roux-en-Y gastric bypass’, ‘LRYGB’, ‘rLRYGB’, ‘single anastomosis duodeno-ileal bypass, ‘single anastomosis loop duodenal switch’, “stomach intestinal pylorus sparing”, “stomach intestinal and pylorus sparing”, “SADI”, “SIPS”. The following MeSH terms were also used: Anastomosis Roux-en-Y and Biliopancreatic Diversion. All these terms were combined using OR or AND. The search algorithms are provided in Appendix 1. References of the selected articles were screened to identify potentially relevant studies, missed by the search algorithm (cross-referencing). Two authors (TT and GRJ) performed the research independently, reviewing all abstracts and relevant full-text articles.

### Inclusion and exclusion criteria

**Fig. 1:**
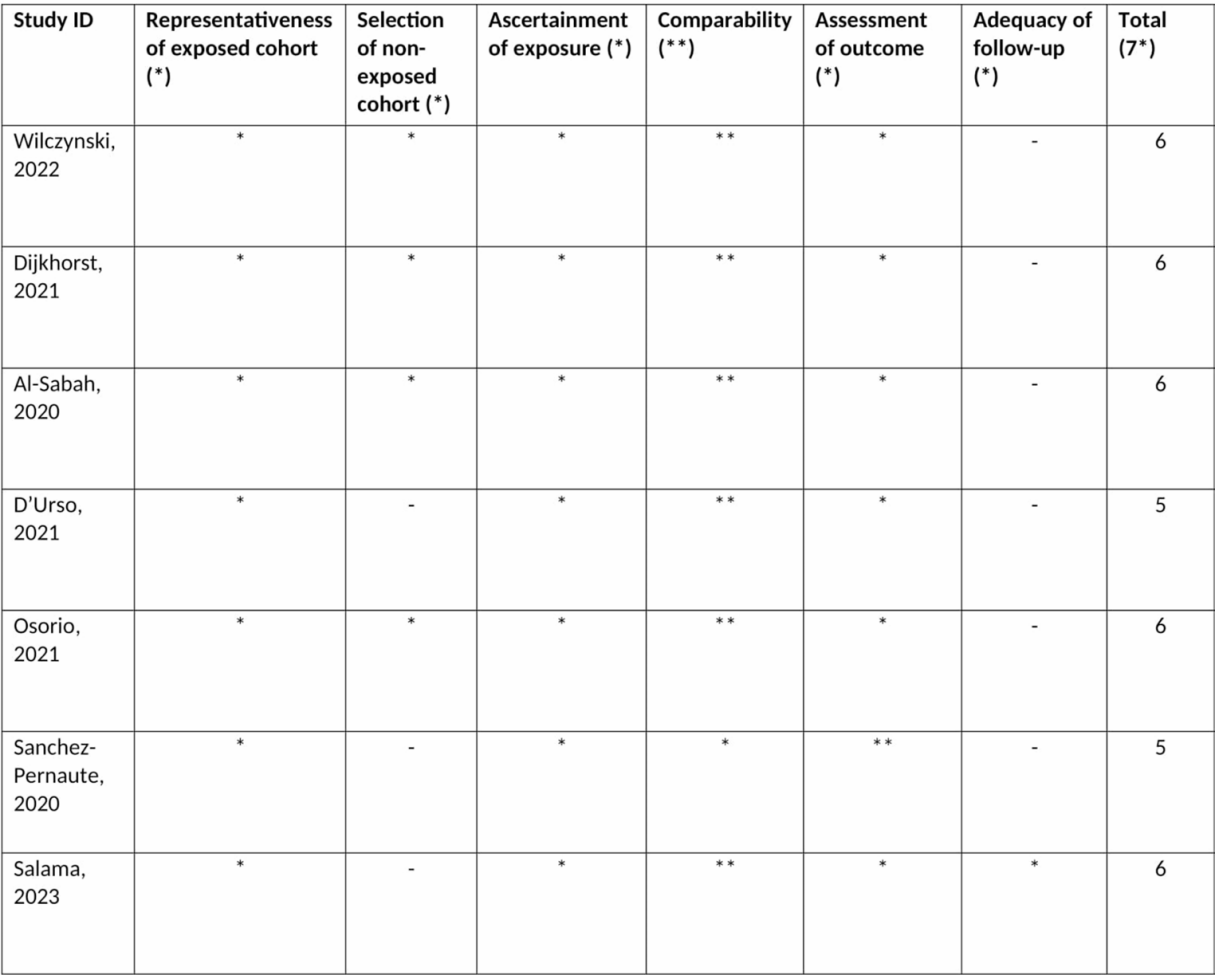
Quality assessment of studies using a modified Newcastle-Ottawa scale for assessing studies in the narrative review of SADI versus RYGB after failed SG

Original studies, comparative and non-comparative, retrospective or prospective in nature were included if SADI or RYGB were performed as RBS after ‘failed SG’. There is no universally accepted definition for what constitutes unsatisfactory outcomes after bariatric surgery, as significant improvement of comorbidity and quality of life is often observed despite suboptimal weight loss. Mann et al. [26], in their systematic review to define indications for RBS, report the inconsistency of definitions of ‘failure’ of a primary bariatric procedure, as well as the lack of clearly defined criteria for RBS. In a general manner, indications for RBS include insufficient weight loss, poor functional outcomes, or both. Insufficient weight loss is defined as an excess weight loss (EWL %) < 50% and/or a residual BMI > 35 kg/m^2^ at 18 months postoperatively [26], according to the NIH consensus guidelines [27] and the 1982 Reinhold criteria [28]. In the particular case of patients with a BMI > 50 kg/m^2^, Biron et al. [29] defined as insufficient weight loss a residual BMI > 40 kg/m^2^, without a time frame specified. Complication-related indications for RBS include GERD, recurrent gastric or anastomotic stenosis, fistulas or intractable anastomotic ulcers [26]. In the present review, both weight- and complication-related definitions of ‘failed’ SG were included. The Ottawa-Newcastle score [30] was used to grade the quality of observational studies, with the cut-off for inclusion set at five points (Fig. 1).

Studies assessing planned two-staged procedures (SADI or BPD-DS) after SG were excluded. In addition, exclusion criteria were experimental (non-human) studies, case and technical reports, series with < 30 included patients, series with follow-up < 5 years, studies referring to pediatric/adolescent population, previous systematic reviews and meta-analyses, editorials, conference abstracts and comments/letters to the editor.

## Study endpoints

The primary endpoint was long-term (≥ 5 years) weight loss after RBS; this was defined as the percentage of total weight loss (TWL %) or percentage of excess weight loss (EWL %), from the value before redo-surgery or since baseline. As secondary endpoints were assessed the duration of the surgery, length of hospital stay, severe functional problems after SG, evolution of obesity-related comorbidities and postoperative complications, graded according to the Clavien-Dindo classification [31].

### Description of surgical procedures

#### Single-anastomosis duodeno-ileal bypass (SADI) [Image 1]

At the very beginning of the surgery, an assessment of the hiatus and hiatoplasty is performed when needed [32]. Afterwards, notwithstanding the technical variations, the surgery consists of creating a loop anastomosis between the first portion of the duodenum, which is transected, and the ileum. The anastomosis may be done with a hand-sewn [32–34] or a stapled-technique [32, 34], and the length of the common channel may vary between 250 and 300 cm [32, 34].

**Image 1:**
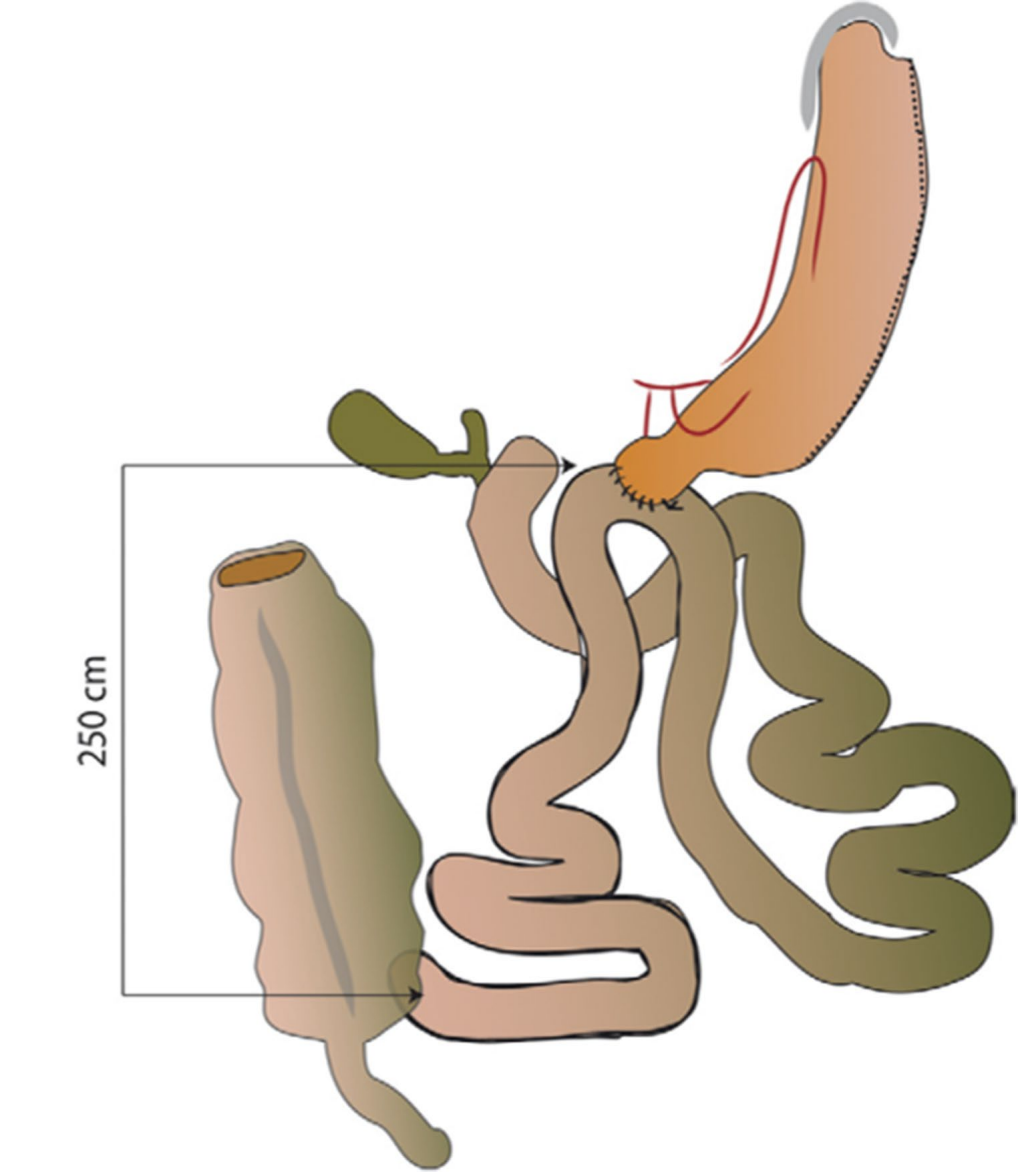
Conversion SG to SADI

#### Roux-en-Y gastric bypass (RYGB) [Image 2]

Conversion from SG to RYGB, for all included studies, was done with the Lonroth technique [35]. The surgery was initiated with a hiatal hernia repair, if indicated [36]. A narrow gastric pouch was created approximately 3–4 cm from the gastroesophageal junction, and the gastro-jejunal anastomosis was conducted with either a linear [37, 38] or a hand-sewn manner [36]. The lengths of the alimentary and biliopancreatic limbs vary between 100 and 150 cm [36, 39, 40] and 70–150 cm [36, 39, 40] respectively.

**Image 2:**
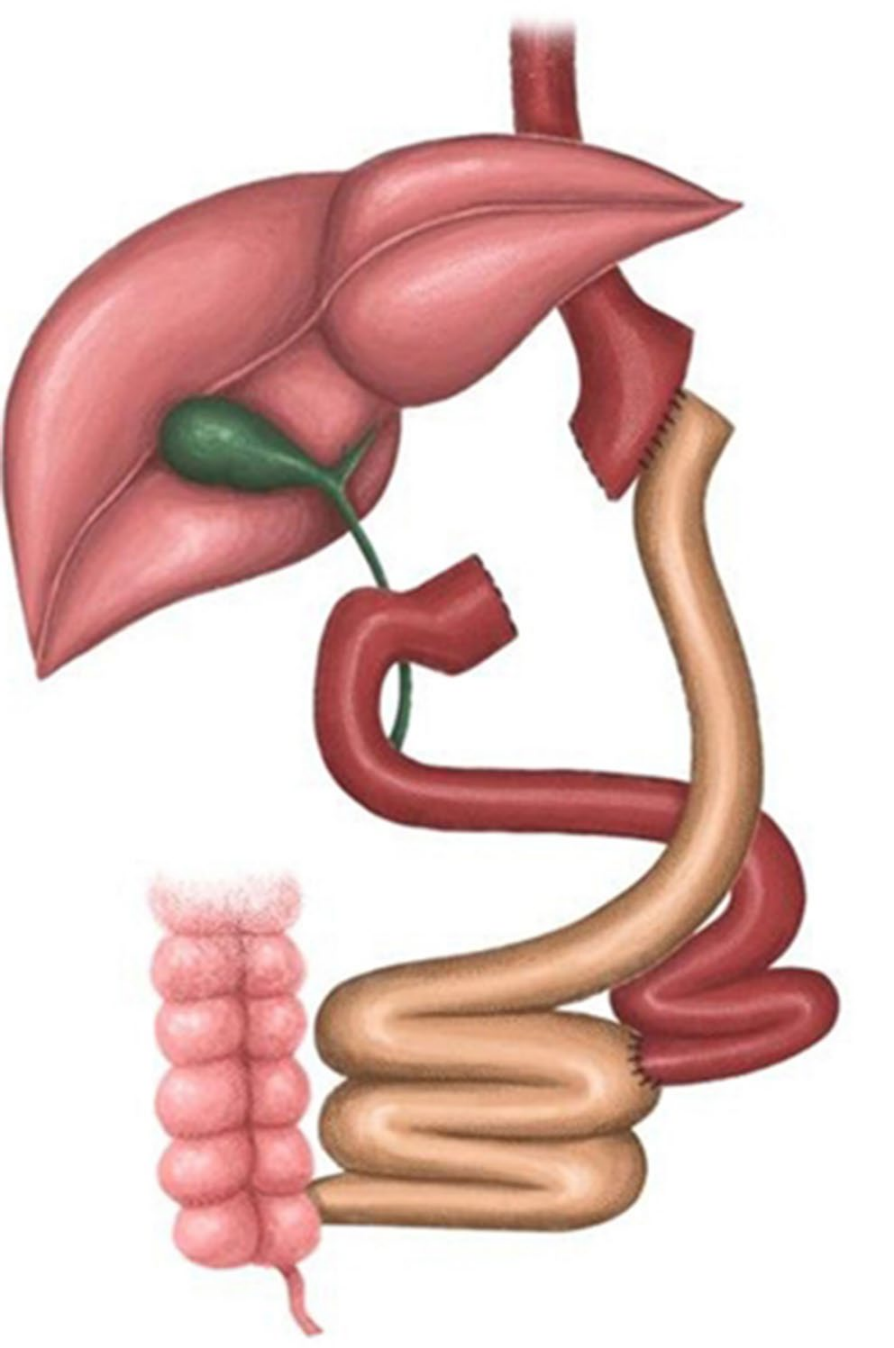
Conversion SG to RYGB

### Statistical analysis

For each endpoint studied, a pooled analysis of available results was performed, providing mean (SD) or median (range) for continuous variables and frequency (%) for discrete ones. Dedicated Excel spreadsheets were used for data collection and analysis. Quantitative synthesis of the results (meta-analysis) was not performed, due to the variability in outcome reporting measures rendering a statistical summary non-reliable.

## Results

**Fig. 2:**
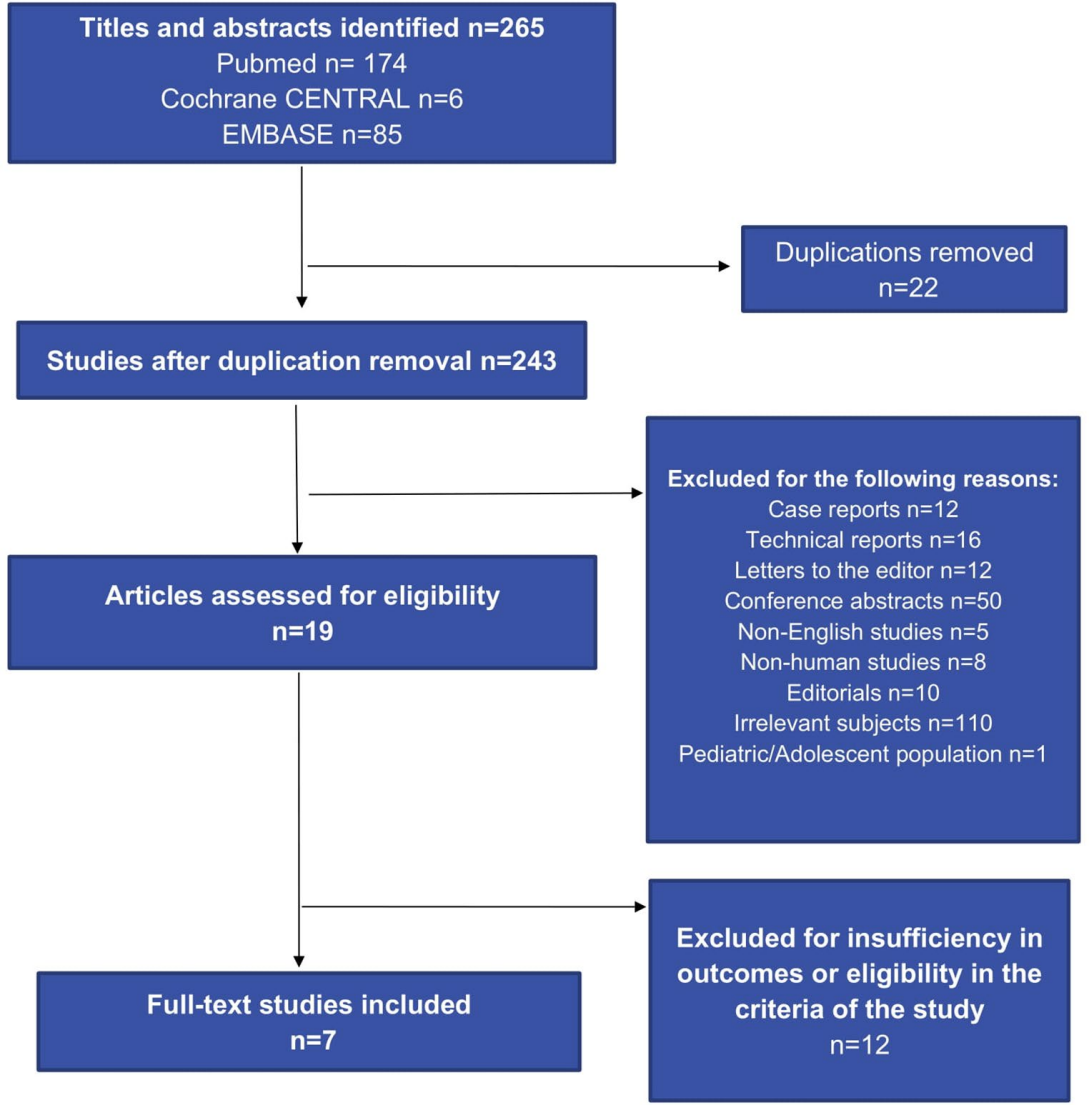
Study’s flowchart

From the 265 search results initially retrieved, seven studies met the inclusion criteria [32–34, 36, 39–41], as illustrated in the study’s flowchart (Fig. 2). There was one comparative study between RYGB and SADI, three including only SADI and three only RYGB as revisional procedures after ‘failed’ SG. All seven studies fulfilled the Ottawa-Newcastle inclusion criteria, as shown in Fig. 1 Indications for RBS included insufficient weight loss (EWL < 50% or residual BMI ≥ 35 kg/m^2^) for all included studies [32–34, 36, 39–41], but also intractable GERD and dysphagia, the latter especially when RYGB was chosen as a revisional procedure [32, 36, 39, 40].

Table 1 summarizes demographics and postoperative outcomes for all included studies. Overall, 419 patients operated between 2007 and 2024 were assessed. The majority (80%) were female, with a median age of 41.7 [range 18–62] years. Median baseline BMI was 51.8 kg/m^2^ [range 37–71 kg/m^2^] before SG, and 40.3 kg/m^2^ [range 30–54 kg/m^2^] before RBS. The median interval between SG and RBS was 44 months [range 4-142 months], and patients had a median follow- up of 48 months [range 6-165 months] after RBS.

As described before, there are no universally accepted criteria for the definition of a ‘failed’ SG, but most of the authors determine the failure as insufficient weight loss, severe functional problems or both. For a better interpretation of our results, we need to mention the same inconsistency in the studies included in our review. All of them [32–34, 36, 39–41] agree on insufficient weight loss using NIH consensus guidelines [27] or the 1982 Reinhold criteria [28]. Moreover, severe functional problems such as intractable GERD or anatomical issues (twist) are described in four studies [32, 36, 39, 40], as well as no resolution of major comorbidities (diabetes, hypertension etc.) in three studies [33, 34, 41].

**Table 1:** Demographics and postoperative outcomes after Revisional SADI and RYGB of included studies

Study	Technique	No. Patients	Age (years)	Operative duration (min)	LOS (days)	Severe postoperative complications* (%)
Sanchez-Pernaute, 2020	SADI	51	42 (18–62)	-	-	2%
Osorio, 2021	SADI	46	45.3 (± 10.6)	-	2.24 ± 1.2	6.5%
Dijkhorst, 2021	SADI Vs RYGB	63 Vs 78	43.6 (± 10.6) Vs 46 (± 11.1)	83.5 [38–199] Vs 75.5 [39–212]	1 (1–8) Vs 2(1–25)	14.3% Vs 20.5%
Salama, 2023	SADI	42	38.0 ± 9.0	-	-	4.7%
D’Urso, 2021	RYGB	60	41 (± 10.1)	-	2.6 (1–6)	25%
Wilczynski, 2022 Al-Sabah, 2020	RYGB RYGB	33 46	41.2 (± 8.9) 37 (± 10.7)	- -	- -	6% 2.4%

RYGB = Roux-en-Y gastric bypass, SADI = single anastomosis duodeno-ileal bypass, LOS = length of hospital stay

Continuous variables are expressed as median [range] or mean (± SD)

*Complications grade III-IV according to Clavien -Dindo classification

### Weight loss results after RYGB and SADI as RBS procedures

Weight loss was reported either as %TWL (32, 33, 34, 39–40, 41) or as %EWL (33–34, 36, 39, 40, 41) in the included studies (Table 2).

**Table 2:** Weight-loss outcomes for revisional SADI and RYGB after failed SG

Study	Indication	Technique	Baseline BMI (before SG)	BMI before revision	Resolution of GERD	BMI at last FU	%EWL or %TWL compared to baseline^1^ or after SG^2^ at 5 years FU
Sanchez-Pernaute, 2020	Insufficient weight loss	SADI	52 (37–71 range)	40.4 (30-53.7 range)	-	30	79%^1^ or 41%^1^, 64%^2^ or 26%^2^
Osorio, 2021	Insufficient weight loss	SADI	56.15 (± 8.5)	39.2 (± 6.2)	-	-	7.57%^2^ or 31.4%^2^
Dijkhorst, 2021	Insufficient weight loss or functional problems	SADI Vs RYGB	53.1 (± 9.7)	44.9 (± 6.2) Vs 39.1 (± 8)	90% (RYGB patients)	-	15% (± 22.8)^2^ Vs 2.1% (± 13.2)^2^
Salama, 2023	Insufficient weight loss	SADI	50 (± 8)	45.9 (± 10.3)	-	33.7 (± 5.8)	66.2% (± 21.7)^2^ or 30% (± 18.4)^2^
D’Urso, 2021	Insufficient weight loss or functional problems	RYGB	45.9 (± 7.4)	38.1 (± 7.1)	90%	-	62.7% (± 22.7)^1^ or 31.1%^1^ or 18.1%^2^
Wilczynski, 2022	Insufficient weight loss or functional problems	RYGB	-	38.7 (± 6.84)	40	-	72.95% (± 20.3)^1^ or 18.4% (± 11.85)^2^
Al-Sabah, 2020	Insufficient weight loss or functional problems	RYGB	50.6 (± 10.4)	42.67 (± 6.85)	90%	34.3(± 5.9)	49.2%^2^

FU = follow-up, RYGB = Roux-en-Y gastric bypass, SADI = single anastomosis duodeno-ileal bypass, SG = sleeve gastrectomy, BMI = body mass index (Kg/m2), EWL = excess weight loss, TWL = total weight loss

Continuous variables are expressed as median [range] or mean (± SD)

EWL% in blue, TWL% in red

Data concerning weight loss after revisional RYGB present a high heterogeneity because of the different design of included studies [36, 39, 40]. D’Urso et al. [40] reported 62.7% EWL and 31.1% TWL from baseline and 18.1% TWL since revisional surgery, but with a follow-up rate of only 55% at 5 years. Wilczynski et al. [39] reported 73% EWL from baseline and 18.4% TWL from RBS, with a loss to follow-up after 5 years of almost 75%. Al Sabah et al. [36] reported 49.2% EWL after 5 years in the RYGB group, with a complete follow-up rate of 24%. BMI values at the last follow-up were reported only in this study with a mean value of 34.3 kg/m2 and a BMI loss of 8.3 units since RBS.

Reports of weight loss results after SADI were more homogenous; SADI offered a median 24.5% TWL [range 19.3–44.5%] after RBS [32–34, 41]. In the study by Sanchez-Pernaute et al. [34], the total SADI cohort had a mean TWL of 26% and a EWL of 64%, as well as the reduction of the BMI by 11.5 units after RBS and 22 from baseline. The mean EWL% and TWL% were, respectively, 79% and 39% at 1 year, 81% and 41% at 2 years, 76% and 38% at 3 years, 80% and 41% at 4 years, and 79% and 41% at 5 years after SADI.

Osorio et al. [33], reported a %TWL of 31.4% after RBS (or EWL 57.57%) at 5 years, but with only a nearly 24% of follow-up at this time point. Compared with the classic duodenal switch, SADI patients presented a significant weight regain beginning in the second post-operative year (difference + 11.2% EWL), and more evident at 5years (difference + 12.9%EWL).

Asaad et al. [41] reported 66.2% EWL and 30% TWL after SADI and since revisional surgery, with a follow-up rate of more than 90% at 5 years. Notably, there was a marked reduction in BMI 5 years after undergoing SADI, from an average of 45.9 ± 10.3 to 33.7 ± 5.8 (p value < 0.001). Moreover, compared to one-anastomosis gastric bypass in that particular cohort of patients, the weight assessment variables measured 5 years post surgery, including the difference in BMI, TWL%, and EWL%, all showed statistically significant differences in favor of the SADI procedure.

Dijkhorst et al. [32] in the only comparative study, concluded that the SADI resulted in significantly more weight loss at 5 years (%TWL: 19.8 SADI versus 8.1 RYGB, *P* <.001). More precisely %TWL following revisional surgery at 1, 2, 3, 4, and 5 years was 22%, 24%, 22%, 18%, and 15% for SADI-S and 10%, 9%, 7%, 8%, and 2% for RYGB. 72% of RYGB patients presented a weight-loss plateau between the 1st-2nd years after RBS as previously suggested by other authors [42] and 78% never reached an EWL > 50%. On the other hand, SADI patients seemed to lose progressively weight during the first 2 years, although they presented a mean TWL < 20% after 3 years of follow up.

### Postoperative complications and functional outcomes

Operative time was mentioned only in one study [32], preventing a safe comparison between the two procedures. Median length of stay, cited in 3 studies, was 2.3 days [range 1–25 days] for RYGB [32, 40], and 1.6 days [range 1–8 days] for SADI [32]. Severe (Grade III-IV) complications occurred in 8% (range 2–14%) of patients after SADI [32] and 14% after RYGB (range 2.4–25%) [32, 36, 39, 40]. In the only comparative study included in our review, no significant differences were found between RYGB and SADI patients, in terms of short- or long-term severe complication rates, as well as post-operative quality of life and average physical health score [32].

Resolution of functional problems after SG, such as GERD or dysphagia, is reported in four studies [32, 36, 39, 40]. RYGB is described as a salvage procedure of choice after SG in this indication, resulting in complete GERD resolution and significant improvement of quality of life in up to 90% of patients [32, 36, 40]. One study reported complete remission of GERD in 40% of patients, and improvement of symptoms and esophagitis in 60% of patients after RYGB [39] (Table 2).

### Comorbidity resolution after RYGB and SADI as RBS

Five studies reported long-term evolution of comorbidities after RBS (33–34, 36–37. 51). Osorio et al. [33], in a series of SADI patients, reported resolution rates of 44.4% for diabetes, 36.4% for hypertension, 72.7% for dyslipidemia and 93.3% for obstructive sleep apnea. Sanchez-Pernaute et al. [34] reported a remission rate of 56% for hypertension, 94% for diabetes and 74% for dyslipidemia after SADI. Wilczynksi et al. [39] found a low long-term diabetes and hypertension remission of 33% and 30% respectively, on the contrary 83.3% of dyslipidemia and 100% for obstructive sleep apnea in the RYGB group. In another study comparing re-sleeve with conversion to RYGB, Al Sabah et al. [36] found a remission rate of 38.5% for hypertension, 22.2% for diabetes and 100% for dyslipidemia after RYGB. Finally yet importantly, Asaad et al. [41] reported 75% of resolution of diabetes, while the remaining 25% of patients demonstrated a reduction in their anti-diabetic medications, as well as 50% resolution of hypertension and cessation of the anti-hypertensive medications.

## Discussion

The present review is the first in the literature to report long-term (≥ 5 years) results of SADI and RYGB as RBS after ‘failed’ SG. Weight regain or insufficient weight loss was the primary indication for RBS after SG. We found that SADI compared to RYGB, showed increased weight loss after failed SG over time, although the difference between the two was significant in just one study [32]. On the other hand, in cases of invalidating GERD or dysphagia after SG, RYGB was the procedure of choice, with symptom resolution in up to 90% of patients [14, 15].

In parallel to the worldwide increase of SG in recent years, RBS after SG is also on the rise, mainly due to insufficient weight loss or functional problems such as gastroesophageal reflux disease, gastric stricture and dysphagia [43]. The exact incidence of ‘failed’ primary SG, referring to suboptimal weight loss, poor functional outcomes or both, is difficult to assess but increases over time, reaching up to 25% 10 years after surgery in some cohorts [8, 37]. It is still a matter of debate whether RYGB, re-SG, BPD/DS or SADI is the best redo-surgery in this situation. The most appropriate revisional procedure depends on the main indication for RBS, and needs a thorough multidisciplinary assessment of the patients, including endoscopy and imaging to define the exact anatomy of the primary operation. Previously published data suggested that highly malabsorptive procedures, such as the BPD/DS or SADI may offer better weight loss compared to RYGB, at least in the short-midterm [38, 44]. However, the risk of serious nutritional deficiencies must be taken into account when considering these procedures [45].

Proponents of SADI advocate that it is associated with fewer marginal ulcers, anastomotic leaks and anastomotic strictures, a negligible internal hernia risk, and reduced nutritional and protein deficiencies compared to the classic BPD/DS operation [44, 46]. Due to this safety profile, SADI is now an accepted bariatric-metabolic procedure within IFSO Guidelines (International Federation for the Surgery of Obesity and Metabolic Disorders) [47]. However, mid- and long- term data about its efficacy are scarce in the literature, with even less evidence existing in the context of RBS [48].

A recent matched analysis of SADI vs. RYGB as primary procedures, showed that long-term (≥ 5 years) complications, reinterventions, suboptimal weight loss and conversion, were more frequent after RYGB [49]. In particular, Landrenau et al. [50] showed a 31.5% complication rate, Boru et al. [51] reported 10% of complications and Ianelli et al. [42] reported 16.7% of post-operative complications after RYGB.

Short/mid-term results of SADI versus RYGB have been reported in two systematic reviews; Lee et al. [24] reported that the SADI/BPD-DS group achieved a significantly higher percentage (by 10.2%) of TWL compared to RYGB, with no significant differences in adverse events, or improvement of co-morbidities between the two groups. The most important limitations were the significantly higher initial BMI in the SADI/BPD-DS group, as well as the lack of subgroup analysis of the SADI data. On the other hand, Chierici et al. [25] showed mean TWL of 28.4% in the BPD-DS group and 19.1% in the SADI group compared to 7.3% in the RYGB group at 3 years of follow-up, with a higher morbidity for the BPD-DS and RYGB. Both of these papers concluded that RYGB and SADI after SG are safe RBS options without significant differences, at least in the mid-term. The two main limitations of these two meta-analyses are small sample size (< 20 patients) in most of the included studies, and their focus on short/mid-term results (12 to 36 months). In contrast, our review has the advantage of an included series with at least thirty patients, as well as the long-term results after RBS with follow-up of at least five years.

Nevertheless, our study has some limitations that need to be considered. First, we note the presence of only one comparative cohort study, and the complete lack of randomized controlled trials on this particular subject. Second, the small sample size, the heterogeneity and the differences in initial BMI between the two groups (with SADI patients having higher baseline BMI) can bias the results, and underestimate the weight loss potential of SADI. Third, the inconsistent definition in the literature of the ‘failed’ SG (weight loss failure or related complications) could eventually lead to bias, without a more thorough and careful interpretation of the results. Finally, a longer follow-up is needed to assess long-term nutritional deficiencies and functional outcomes after RBS in this challenging group of patients.

## Conclusion

The present review found that SADI seems to be a promising and suitable method for suboptimal weight loss after SG, with comparable or even better results to RYGB after 5-year follow-up. On the other hand, conversion to RYGB seems indicated for GERD or other functional problems after SG. However, there is a paucity of high quality long-term data to determine which type of intervention is the most appropriate RBS option in patients with poor weight loss or functional results after SG. Very importantly, caution is needed when defining ‘poor’ results of bariatric surgery, and determining the expected benefit of RBS for these patients. More prospective studies are warranted, in order to help bariatric specialists deal with the challenging, and increasingly numerous, group of patients with suboptimal weight and/or functional results after primary SG.

## Data Availability

No datasets were generated or analysed during the current study.

## Appendix 1

Search strategies.

Ovid MEDLINE(R) ALL 1946 to September 2024.

1013 results on September2024.

(sleeve ADJ3 (gastrectom* OR gastric)).ab, ti, kf. AND (Recidivism/ OR reoperation/ OR exp Treatment Failure/ OR Weight Gain/ OR (fail* OR revision* OR reoperat* OR “re operat*” OR unsuccess* OR unsatisf* OR inefficacy OR recidiv* OR conversion OR converted OR (weight ADJ4 (insufficient OR regain))).ab, ti, kf.) AND (Gastric Bypass/ OR Anastomosis, Roux-en-Y/ OR Biliopancreatic Diversion/ OR (Roux OR RYGB OR LRYGB OR RLRYGB OR ((single OR one) ADJ3 anastomosis ADJ3 duoden*) OR “Duodeno ileal bypass” OR “stomach intestinal pylorus sparing” OR “stomach intestinal and pylorus sparing” OR “loop duodenal switch” OR “loop DS” OR ((biliopancreatic OR “bilio pancreatic” OR pancreatobiliary) ADJ1 (diversion* OR bypass*)) OR SADI OR SADS OR OADS OR SIPS).ab, ti, kf.) NOT (Editorial or Letter).pt.

Embase.com.

1250 results on September 2024.

(‘sleeve gastrectomy’/exp OR ‘gastric sleeve’/de OR (sleeve NEAR/3 (gastrectom* OR gastric)): ab, ti, kw) AND (‘recidivism’/de OR ‘reoperation’/de OR ‘treatment failure’/de OR ‘body weight gain’/de OR (fail* OR revision* OR reoperat* OR “re operat*” OR unsuccess* OR unsatisf* OR inefficacy OR recidiv* OR conversion OR converted OR (weight NEAR/4 (insufficient OR regain))): ab, ti, kw) AND (‘roux y anastomosis’/exp OR (Roux OR RYGB OR LRYGB OR RLRYGB OR ((single OR one) NEAR/3 anastomosis NEAR/3 duoden*) OR “Duodeno-ileal bypass” OR “stomach intestinal pylorus sparing” OR “stomach intestinal and pylorus sparing” OR “loop duodenal switch” OR “loop DS” OR ((biliopancreatic OR “bilio pancreatic” OR pancreatobiliary) NEXT/1 (diversion* OR bypass*)) OR SADI OR SADS OR OADS OR SIPS): ab, ti, kw) NOT (‘conference abstract’/it OR ‘conference review’/it OR ‘editorial’/it OR ‘letter’/it).

Cochrane Central Register of Controlled Trials Issue 6 of 12, September 2024.

97 results on September 2024.

(sleeve NEAR/3 (gastrectom* OR gastric)): ab, ti, kw AND (fail* OR revision* OR reoperat* OR “re operat*” OR unsuccess* OR unsatisf* OR inefficacy OR recidiv* OR conversion OR converted OR (weight NEAR/4 (insufficient OR regain))): ab, ti, kw AND (Roux OR RYGB OR LRYGB OR RLRYGB OR ((single OR one) NEAR/3 anastomosis NEAR/3 duoden*) OR “Duodeno ileal bypass” OR “stomach intestinal pylorus sparing” OR “stomach intestinal and pylorus sparing” OR “loop duodenal switch” OR “loop DS” OR ((biliopancreatic OR “bilio pancreatic” OR pancreatobiliary) NEXT/1 (diversion* OR bypass*)) OR SADI OR SADS OR OADS OR SIPS): ab, ti, kw.
